# Comparison of a group-delivered and individually delivered lifestyle-integrated functional exercise (LiFE) program in older persons: a randomized noninferiority trial

**DOI:** 10.1186/s12877-018-0953-6

**Published:** 2018-11-06

**Authors:** Carl-Philipp Jansen, Corinna Nerz, Franziska Kramer, Sarah Labudek, Jochen Klenk, Judith Dams, Hans-Helmut König, Lindy Clemson, Clemens Becker, Michael Schwenk

**Affiliations:** 1 Network Aging Research, Heidelberg University, Heidelberg, Germany; 2 Department of Clinical Gerontology and Geriatric Rehabilitation, Robert Bosch Hospital, Stuttgart, Germany; 3 Institute of Epidemiology and Medical Biometry, Ulm University, Ulm, Germany; 4 IB Hochschule Berlin, Studienzentrum Stuttgart, Stuttgart, Germany; 5 Department of Health Economics and Health Services Research, University Medical Center Hamburg-Eppendorf, Hamburg, Germany; 6 Faculty of Health Sciences, University of Sydney, Sydney, Australia

**Keywords:** Fall prevention, Functional exercise, Randomized noninferiority trial, Strength, Balance, Physical activity, Health behaviour intervention

## Abstract

**Background:**

The Lifestyle-Integrated Functional Exercise (LiFE) program is effective in improving strength, balance, and physical activity (PA) while simultaneously reducing falls in older people by incorporating exercise activities in recurring daily tasks. However, implementing the original LiFE program includes substantial resource requirements. Therefore, as part of the LiFE-is-LiFE project, a group format (gLiFE) of the LiFE program has been developed, which will be tested regarding its noninferiority to the individually delivered LiFE in terms of PA-adjusted fall incidence and overall cost-effectiveness.

**Methods:**

In a multi-centre, single-blinded noninferiority trial, an envisaged sample of *N* = 300 participants (> 70 years; faller and/or confirmed falls risk; community-dwelling) will be randomized in either LiFE or gLiFE. Both groups will undergo the same strength and balance activities as well as PA promotion activities and habitualization strategies as described in the LiFE programme, however, based on different approaches of delivery: During the 6-month intervention phase, LiFE participants will receive seven home visits and two telephone calls; in gLiFE, the program will be delivered in seven group sessions and also two telephone calls. Main outcomes are a) fall incidence per PA and b) incremental cost-effectiveness ratio comparing costs and quality-adjusted life years between the two interventions. Secondary outcomes include PA behaviour, motor performance, health status, psychosocial status, program evaluation, and adherence. Measurements will be conducted at baseline, 6-month and 12-month follow-up; evaluation of intervention sessions and assessment of psychosocial variables related to execution and habitualization of LiFE activities will be made during the intervention period as well.

**Discussion:**

Compared to LiFE, we expect gLiFE to (a) reduce falls per PA by a similar rate; (b) be more cost-effective; (c) comparably enhance physical performance in terms of strength and balance as well as PA. By investigating the economic and societal benefit, this study will be of high practical relevance as noninferiority of gLiFE would facilitate large-scale implementation due to lower resource usage. This would result in better reach and increased accessibility, which is important for subjects with a history of falls and/or being at risk of falls.

**Trial registration:**

ClinicalTrials.gov NCT03462654. Registered on March 12, 2018.

## Background

Being already high in many Western societies, the proportion of older people—and with it, fiscal and political challenges with respect to health care and society—is projected to increase globally [1]. About a third of older people aged 65 or older experience a fall within 1 year [2, 3], and resulting injuries inevitably have significant repercussions on individuals, the health care system, and the community [4]. Falls are among the top five of the leading health conditions associated with disability in populations aged 60 years and older [5]. Due to this strong impact on individuals, research on falls and fall-related outcomes has received intensified attention in the past decades, and still remains in the spotlight of health-related research. Although many risk factors for falling have been identified in previous research, strength, balance, and gait impairments are among the strongest [6], indicating that respective exercise may be effective in reducing risk and rate of falling. Results of meta-analyses suggest exercise to be the best univariate approach to prevent falls at a population level, however, this depends on the exercise component applied [4, 6]. Sherrington and colleagues recommended balance training as the exercise of choice, ideally accompanied by strength training [4]. Adherence to respective exercise recommendations [7] was reported by only 21.0% of retired seniors, with only 5.3% adhering to both forms of exercise [4, 8]. Several structured training programs which aim to enhance balance and muscle strength (e.g., the Otago Exercise Program [9]) are available [4, 10–15]. While such strength and balance exercise programs have been found effective in intervention studies, they often fail to induce long-term change, adherence (> 6 months), and participation [16, 17]. Another, more general factor often brought into play is physical activity (PA). While its numerous health benefits are well established [18], and some of these are connected to a lower risk of falling [19], findings on the relation between PA and risk of falling remain controversial [20–23]. This might be due to increased fall risk exposure, that is, occurrence of situations associated with falls. As up to one half of falls in the 65+ age group occurs while walking [24, 25], walking duration may be an adequate surrogate for risk exposure time [26]. Despite the large number of older adults not meeting evidence-based PA guidelines [27], and given the popularity of walking activities in older cohorts, such recommendations for regular PA may have to be followed with caution when it comes to the adoption and promotion of PA in subjects with moderate to high fall risk. It may not be sufficient to simply adopt a more active lifestyle; the physical and functional foundation should be established for a “safe gain” in physically active behaviour.

Novel concepts and formats with the potential for large-scale implementation and long-term adherence to strength and balance exercising are urgently needed. With the ‘Lifestyle-integrated Functional Exercise’ (LiFE) program [28], Clemson and colleagues presented a novel approach to prevent falls by improving strength and balance while simultaneously promoting the adoption of a physically more active lifestyle in persons aged 70 years and older. The integration of the LiFE activities is assumed as a gateway behaviour to more PA, meaning that through practicing the functional balance and strength exercises, PA behaviour is enhanced. Unlike structured exercise programs, LiFE promotes the idea to incorporate balance and strength activities into everyday tasks rather than participating in a structured exercise program at certain occasions. In a randomised controlled trial, LiFE was found superior to a structured group exercise and a control program in improving physical function, reducing functional disability, promoting adherence, and enhancing PA while significantly reducing falls [29]. Notwithstanding its effectiveness, implementing LiFE as a home-based program poses high financial requirements and human resources. Program delivery requires seven individual home visits and two follow-up phone calls, in which participants are taught in a one-to-one training how to successfully implement LiFE activities into their personal daily routine. Very recent findings suggest that LiFE may be as effective when delivered in a group setting as compared to the individual approach [30]. Still, an evaluation of a LiFE group format based on a larger sample is missing and warranted [30]. Therefore, as part of the LiFE-is-LiFE project, the original, individually delivered LiFE program was adapted into a group format (gLiFE) with the aim to facilitate large-scale implementation and thus less resource usage. The adapted approach delivered in the trial at hand (gLiFE) was tested in a small pilot study (study registration ID: NCT03412123; concept paper under preparation). In this current LiFE-is-LiFE project, gLiFE is going to be tested for its noninferiority compared to LiFE in terms of fall incidence and cost-effectiveness.

### Aims

We hypothesize in this multi-centre, two armed, single-blinded, randomised noninferiority trial that: *(1)* gLiFE won’t be less efficacious than LiFE in terms of reducing fall incidence expressed as number of falls per PA, i.e., energy expenditure; gLiFE won’t result in a lower intervention retention rate (i.e., percentage of the sample completing the 6-month and 12-month follow-up assessment) as compared to LiFE; *(2)* delivering gLiFE will be cost-effective and less costly compared with LiFE; *(3)* in both groups, physical performance in terms of strength and balance as well as physical activity will be enhanced at comparable levels.

A process evaluation according to guidelines of the Medical Research Council (MRC) for complex interventions [31] will be performed in order to assess quality of implementation and to identify causal mechanisms as well as contextual factors which may affect study outcomes.

## Methods/design

### Study design and setting

This multi-centre, single-blinded noninferiority trial is designed in accordance with the extended CONSORT statement for reporting on noninferiority trials [32]. *N* = 300 participants living in the respective communities are going to be recruited from two study sites (*n* = 150 at each site): The Network Aging Research (Heidelberg, Germany) and the Robert Bosch Hospital (Stuttgart, Germany). This protocol was drafted following the SPIRIT guidelines for randomized trials [33].

### Eligibility criteria

German-speaking, community-dwelling seniors aged 70 years and older being able to walk at least 200 m with or without walking aid will be eligible for participation if they either a) experienced at least one injurious fall within the past year, or b) experienced more than one non-injurious fall within the past year, or c) stated having perceived a balance decline within the past year and needed ≥12 s for the “Timed Up-and-Go” [34] test. In- and exclusion criteria are presented in Table 1; they were chosen in accordance with those defined in the randomised trial by Clemson et al. [29] to ensure external validity of the trial.

**Table 1:** Overview of eligibility criteria

Inclusion criteria	Exclusion criteria
- Age: ≥ 70 years	- Exercise > 1/week in past 3 months - Moderate to vigorous physical activity > 150 min/week in past 3 months
- Living at home *OR* “supervised living” without having active assistance	- Medical conditions: - Heart failure (NYHA class III & IV) - Recent cerebrovascular accident (< 6 months) - Parkinson’s disease - On active cancer treatment (last 6 months) - Chronic Obstructive Pulmonary Disease Gold class III & IV - Unstable lower limb fracture - Amputated lower extremity (−ies) - Acute treatment of depression - Uncontrolled resting blood pressures of a systolic > 160 or diastolic > 100 or higher
- Fall risk, defined as > 2 falls within the last 12 months *OR* 1 injurious fall within the last 12 months *OR* subjectively perceived balance decline AND Timed Up-and-Go time ≥ 12 s	
- Able to speak and read in German	
- Able to ambulate 200 m without personal assistance	- Unavailability for home visits within 11 weeks after baseline assessment
	- Travel > 2 months planned within first 6 months of the study
	- Moderate to severe cognitive impairment (Montreal Cognitive Assessment < 23)
	- Current participation in another scientific trial

### Interventions

The two intervention arms (*n* = 150 participants in each arm) are going to contain the same strength and balance activities as well as principles and habitualization strategies as described in the original LiFE program manual [35], but will use different approaches of delivery (i.e., group vs. individual). The intervention sessions in both arms are going to be conducted by physio therapists, occupational therapists and/or sports scientists. Trainers attended a two-day workshop to ensure standardised delivery of all gLiFE and LiFE intervention components and were tested and awarded certification prior to the start of the intervention delivery. A detailed description of the intervention components is provided using the TIDieR checklist [36] (Table 2); a schematic overview of the seven sessions is presented in Table 3.

**Table 2:** Template for Intervention Description and Replication (TIDieR) checklist

Item No.; Name	Description	
1. Brief name	Lifestyle-integrated Functional Exercise (LiFE): individually delivered (LiFE) and group-delivered (gLiFE)	
2. Why	The LiFE program was shown to be effective in reducing falls while at the same time improving balance, strength, and enhancing physical activity. Due to high economic requirements regarding the program’s delivery, a group-based delivery of the program is tested to evaluate whether a more cost-effective approach can be successful.	
3. What: Materials	Participant’s manual, German version [42]; used during and after intervention: Contains descriptions and instructions of all LiFE activities; principles of balance and strength training as well as physical activity enhancement; precautions and safety instructions when performing the activities; background on balance and strength exercise; assistance and support for changing habits and performing LiFE activities. Trainer’s manual, German version; one for LiFE, one for gLiFE. Contains all information also included in the participant’s manual; additionally: complete outline of all 7 sessions and 2 phone calls, including text templates, material, preparations, and precautions. Working book; for all participants; used during intervention: Includes information on study procedures, personnel, contacts, and safety instructions; activity planning sheets for balance, strength, and physical activity; notes pages; LiFE principles; ‘life compass’ LiFE Assessment Tool (modified Version in German); for trainers; used to determine individuals’ performance level of LiFE activities Laminated cards, showing LiFE principles and LiFE activities to be used as visual aids during intervention sessions. Further materials to be used in interventions sessions: balls, blankets, sponge rubber, boxes, clipboards, pens, bags, name tags, flipcharts.	
4. What: Procedures	LiFE 7 home visits by one qualified trainer, 2 telephone calls 4 and 10 weeks after last session.	gLiFE 7 group sessions (n = 8–12 participants) led by one main and one co-trainer, 2 telephone calls 4 and 10 weeks after last session.
	In both intervention arms, LiFE activities, identification of daily situations to integrate activities, their selection, implementation, and upgrading are addressed. In session 1, 4 LiFE activities are introduced; in each subsequent session, 2 other new activities are added. One theoretical lesson is given in each session; topics are: (1) LiFE principles, (2) cues and prompts, (3) upgrading, (4) coping planning, (5) resources for habit formation, (6) mindfulness vs. habit, and (7) long-term success with LiFE. Action planning and implementation intentions are addressed at the end of each session. To compensate for not being in the individual’s home and the lack of knowledge on the person’s environment, in gLiFE, visualization techniques are used to support and facilitate action planning as well as habit formation.	
5. Who provided	Trainers are either sport scientists, physiotherapists, occupational therapists or psychologists. All trainers received a two-day training course on the program background, aims, and components prior to the project start.	
6. How	After randomization, the intervention is provided either in a one-to-one situation in the participant’s home or in a group setting with 8–12 participants.	
7. Where	Two study sites: Heidelberg and Stuttgart (Germany).	
	LiFE Delivered in participants’ homes in suburban Heidelberg area (max. 15 km from the city centre) / one large city district in Stuttgart.	gLiFE Participants attend sessions at the Network Aging Research (Heidelberg) / rented rooms near the recruitment district in Stuttgart.
8. When and how much	LiFE 7 sessions within 11 weeks: week 1, 2, 3, 5, 7, 9, 11. Two telephone calls 4 and 10 weeks after the last session (i.e., week 15 and 21). Duration of each session: 1–1.5 h.	gLiFE see LiFE. Duration of each session: 2–2.5 h.
	Intensity and dose are determined by the individuals’ activity plans, adherence, and performance level of each activity.	
9. Tailoring	In (g)LiFE, irrespective of its method of delivery, individual tailoring is constantly given due to the activities’ integration into the subjects’ personal routine. LiFE activities, their frequency and intensity are determined by the participants and their situation in which the activities are integrated.	
10. Modifications	n.a.	
11. How well: Planned	To assess adherence to i/gLiFE, participants fill out the Exercise Adherence Rating Scale (EARS; [63]) on a monthly basis. Completed forms are then sent to the study centres in Heidelberg and Stuttgart by mail. Activity planning sheets used during the intervention phase also contain check boxes for each day (*activity completed as planned* vs. *not completed as planned*). Fidelity of the intervention delivery is pursed by providing a comprehensive trainer’s manual, in which each intervention session is outlined in large detail to ensure standardized delivery of all intervention components. All trainers have received a two-day training course.	
12. How well: Actual	n.a.

**Table 3:** Structure of the 7 intervention sessions in LiFE and gLiFE

	1	2	3	4	5	6	7
Aim	Introduction	Determination of activities, habit formation and problem solving					
Intro	Familiarization	Repetition of the learned exercises, most positive experiences and greatest challenge in the execution of LiFE					
Main part	LiFE principles	Cues, prompts	Upgrading	Coping planning	Resources for habit formation	Physical activity	Long-term success with LiFE
	LiFE activities: −4 activities (tandem stand, tandem walk, sit to stand, squatting) -practice 2 of them [1 balance/1 strength]	Mediation of two new LiFE activities -practice the activities -keep/skip?					Repetition LiFE activities
		-leaning -walking on toes	-stepping over objects -walking on heels	-walk more -climbing stairs	-tighten muscles -one-leg stand	-move sideways -sit less	
	Action planning/implementation intentions						
End	Assessments, wrap-up & “homework”						

#### Individual LiFE (LiFE)

In LiFE, the program will be taught in seven individual home visits within 11 weeks, as described in the original LiFE program manual [35]. Each home visit is going to take between 1 and 1.5 h. Trainers are going to present a total of five balance activities, seven strength activities for the lower extremities, and two activities to increase general PA are presented. Participants are going to learn how to implement the LiFE activities into their daily routine and how to independently execute the activities, including selection, upgrading, and identification of daily situations to integrate activities. In addition to the home visits, all participants are going to receive two ‘booster telephone calls’ four and 10 weeks after the last intervention session.

#### Group LiFE (gLiFE)

**Fig. 1:**
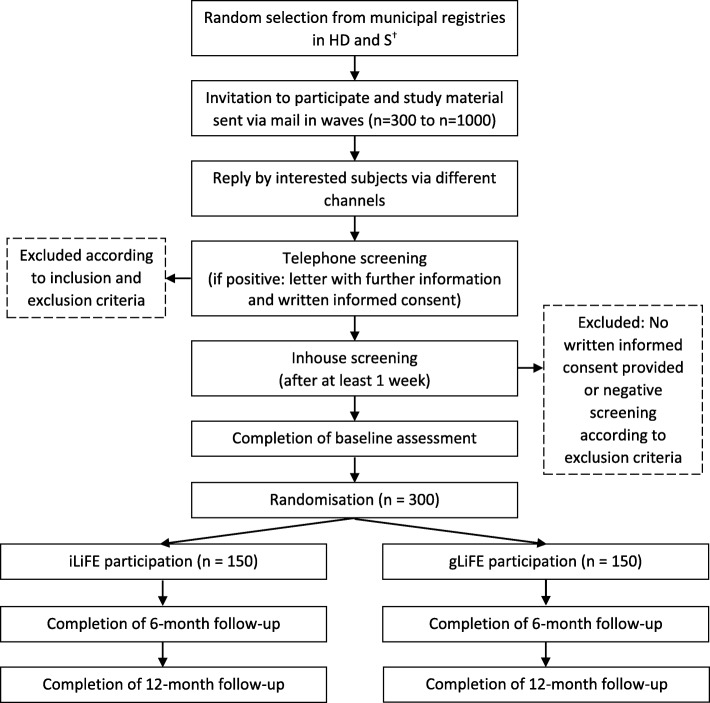
SPIRIT participant flow chart; ^†^in Stuttgart, a health insurance company additionally contacted their members who matched the inclusion criteria

gLiFE will consists of seven group sessions (*n* = 8–12 participants) which are held over the course of 11 weeks, with a duration of about 2 h per session. Each session is going to be led by one main and one co-trainer. In all group sessions, trainers are going to teach the participants how to perform and integrate LiFE activities into their personal daily routine in congruence with LiFE. After the group sessions have ended, participants will also receive two booster telephone calls as in LiFE (see Fig. 1).

Based on predefined criteria by Moore et al. [31], the conceptualisation of the gLiFE program was performed by an interdisciplinary team consisting of sports scientists, psychologists, geriatricians, occupational therapists, and physio therapists in close consultation with one of the originators of the LiFE program (L. Clemson) and the lead investigator of a previous approach to adapt LiFE to a group setting [37]. In addition, the Health Action Process Approach (HAPA) [38]—as an established model to explain health behaviour—was used and extended to support the adaptation of the original LiFE components to the requirements of the group format, with emphasis on intention quality and habit formation [39, 40]. Trainers received a two-day workshop to ensure standardised intervention delivery in both study sites. The final gLiFE concept was piloted in a sample of community-dwelling older adults who matched the criteria of the envisaged sample in the trial. The pilot used a mixed-method approach including quantitative and qualitative (focus group) questions for estimating feasibility and refinement of the intervention before drawing up the final versions of the trainer’s manuals for LiFE and gLiFE. These were drafted following the already existing manual by Clemson et al. [35] to ensure reproducibility of all intervention components and facilitate intervention conduct and organisation. Trainer’s manuals include a complete outline of each session, instruction, and exemplary transcriptions to achieve high fidelity and facilitate uniform implementation. The LiFE participant’s manual [41] has been translated into German language by experts involved in LiFE-related projects [42].

#### Participant safety and adverse events

All study participants will receive specific guidelines for safe training (shoes, support options, lighting, etc., described in the LiFE manuals). Serious adverse events / reactions and adverse events / reactions will be monitored throughout the study to assess the safety of the trial and manage participant risk. Adverse events related or potentially related to study participation will be reported to the responsible Ethic Review Board.

### Outcomes

A complete list of screening and outcome parameters and descriptive variables is provided in Table 4. Data include socio-demographics, medical and medication information, neuropsychological status, motor function, health status and economics, physical activity status, psychosocial data, and information on adherence to LiFE activities and group evaluation. To ensure highest possible standardisation of assessments, instructors attended a two-day workshop covering all aspects of screening and assessments.

**Table 4:** Overview of descriptive variables and assessment measures over the course of the study

		TS	IS	BA	Int	FU6	FU12
	Socio-demography						
	Age; birthday; gender; living conditions (institutionalised vs. community-dwelling)	x					
	Living alone or not; marital status; school-leaving qualification; years of education; academic grades; retirement age		x				
	German-speaking	x					
	Medical and medication information						
	Height; weight		x			x	x
	Blood pressure^f^		x				
	Vision impairment: *Are you able to read a newspaper or book, with or without visual aid?*	x					
	Hearing impairment (whisper test)		x				
	Fall history and fall-related injuries in the past 12 months	x					
P	Fall calendar^d^ [43]						
	Prevalence of neurologic, pulmonary or cardiac disease	x					
	Comorbidities incl. Treatment; cardiac issues or stroke in past 6 months; pain while walking and resting; blood pressure [mmHg]; pulse [1/min]; use of sedatives or anticonvulsants; number of hospital admissions in past 12 months; urinary incontinence in past 12 months		x				
	Medication use (type, dosage, frequency)			x		x	x
	Neuropsychological status						
	Depressive symptoms: Center for Epidemiologic Studies Depression Scale, 10 item Version [CES-D 10] [80]		x				
	Cognitive status: Montreal Cognitive Assessment [MoCa] [81]		x				
S	Fear of falling: Short Falls Efficacy Scale International [Short FES-I] [53]			x		x	x
	Motor function						
	Occurrence of dizziness or gait insecurity in past 12 months		x				
S	Balance self-efficacy: Activities-specific Balance Confidence Scale [ABC-Scale] [58]			x		x	x
S	Functional mobility: (instrumented) Timed Up-and-Go Test [34]	x		x		x	x
S	Functional capacity: Late-Life Function and Disability Instrument [LLFDI] [57]			x		x	x
S	Static balance: 8 Level Balance Scale [29]			x		x	x
S	Static balance: (instrumented) Modified Clinical Test of Sensory Interaction on Balance [54]			x		x	x
S	Static balance: (instrumented) tandem stance with eyes closed			x		x	x
S	Functional leg strength: (instrumented) 30 s chair rise [55]			x		x	x
S	Gait performance: (instrumented) 7 m walking test (usual and fast pace)			x		x	x
S	Handgrip strength: dynamometer			x		x	x
	Health status and economics						
	Subjective health: *Compared with other people in your age group, how would you rate your personal health?*		x				
P	Health-related quality of life: EQ-5D-5 L and EQ-VAS [50]			x		x	x
S	Health-related resource use: adapted version of the questionnaire for the use of medical and non-medical services in old age [FIMA] [59]			x		x	x
	Physical activity status						
	Participation in regular exercise activities > 1/week in the past 3 months; Execution of > 150 min/week moderate to vigorous physical activity	x					
P/ S	Accelerometer-collected physical activity (energy expenditure; duration, percentage, and intensity of sedentariness, activity, and walking)			x		x	x
	Psychosocial questionnaires						
S	Subjective (felt) age: visual analogue scale and years			x		x	x
S	HAPA-related questionnaires: Intention, action and coping planning, individual action control, sources of self-efficacy [60]			x	x^c^	x	x
	HAPA-related questionnaires: outcome expectations, risk perception [60]			x			
S	HAPA-related self-efficacy: motivational, maintenance, recovery [60]			x		x	x
S	Habit strength: Self-Report Behavioural Automaticity Index^e^ [SRBAI] [40]			x	x^c^	x	x
S	Motivational quality: Behavioural Regulation in Exercise Questionnaire [BRE-Q-3] [62]			x			x
S	Social support: Loneliness Scale [61]^e^			x		x	x
	Affiliative Tendency and Sensitivity to Rejection Scale [82]			x			
	Psychological Need Satisfaction in Exercise Scale [83]				x^b,c^		
	Group cohesion: Cohesion in teams–Leisure and health sport [KIT-FG] [84] (only gLiFE participants)^e^				x^a,c^		
	Adherence and group evaluation						
S	Exercise adherence: Exercise Adherence Rating Scale^d,e^ [EARS] [63]				x	x	x
	Evaluation of the intervention session: school grades scale^e^				x^a–c^		
	Satisfaction with the LiFE program^e^				x^c^	x	
	Questions on motivation of participants (only trainers)^e^				x^a–c^		

^a,b,c^*:* session number, after which the respective questionnaire is administered

^d^: part of monthly-returned fall calendar over 12 months

^e^: included in process evaluation

^f^: if > 160 / 90, the person’s general practitioner has to provide consent for participation

*BA* baseline assessment, *FU6* 6 month follow-up, *FU12* 12 month follow-up, *Int* within-intervention assessments, *IS* inhouse screening, *P* primary outcome measure (or part of it), *S* secondary outcome measure, *TS* telephone screening

#### Primary outcome measures

Fall incidence: expressed as number of falls in relation to total energy expenditure. Clinical relevance and sensitivity of this combined index has been validated recently in a cohort of 1214 community-dwelling older adults in Germany [26].

Falls will be assessed based on the fall definition provided by Lamb et al. [43], using an established procedure, i.e., a fall calendar which is returned to the respective study centre on a monthly basis for a period of 12 months. If a person has fallen, information on date, time, injuries and subsequent treatment related to the fall, location of the fall, and movement during which the person has fallen has to be provided on the sheet. In congruence with recommendations [15], a telephone-interview will be performed to rectify missing data, to ascertain details on injuries, and to confirm the current health status of the person. Injurious falls will be categorized according to a standardized system [44].

Physical activity will be assessed using “activPAL4™ micro” accelerometers (PAL Technologies Ltd., Glasgow, Scotland). It is a light, small sized (45 × 25 × 5 mm) triaxial accelerometer worn on the central front thigh over a period of 9 consecutive days (i.e., 7 days with complete data over 24 h). Body posture (sitting/lying, standing/upright) and walking activity are derived from raw data. The device has shown good to excellent reliability [45, 46] and criterion validity in identifying metabolic equivalent of task values [47] as well as body postures [48].

Cost-effectiveness: Cost-effectiveness will be assessed by the incremental cost-effectiveness ratio (ICER) represented by the ratio of the difference in costs and the difference in health effects between both interventions. Costs include in- and outpatient treatment, formal and informal care, transportation and medication as well as intervention costs due to labour costs, room hires, transportation of staff and participants, and material costs. Health effects are measured using quality-adjusted life years (QALYs) based on the EQ-5D-5 L [49, 50]. German values sets for the EQ-5D-5 L have been published recently [51]. The concept of QALYs is commonly used in health economic evaluation for measuring health effects. It combines health-related quality of life with length of life and enables comparing health effects across different diseases [52].

#### Secondary outcome measures

Secondary outcome measures are pointed out in Table 4. Fear of Falling will be assessed using the Short Falls Efficacy Scale International [53]. Motor function will be assessed in terms of instrumented (i.e., smartphone-supported) gait, functional mobility, functional strength and balance tests using the Timed Up-and-Go Test [34], 8 Level Balance Scale [29], Modified Clinical Test of Sensory Interaction on Balance [54], tandem stance with eyes closed, 30-s chair rise [55], and 7 m walking test at usual and fast pace. The smartphone will be worn in an elastic band around the waist during the tests and will provide information on a variety of movement-related parameters, e.g., trunk sway, sit-to-stand duration, jerk during sit-to-stand, and step time [56]. Handgrip strength will be measured with a JAMAR hand dynamometer. Subjective functional capacity will be assessed by the Late-Life Function and Disability Instrument (LLFDI) [57]; balance self-efficacy is assessed using the Activities-specific Balance Confidence Scale [ABC-Scale] [58]. Health-related resource use will be assessed using an adapted version of the questionnaire for the use of medical and non-medical services in old age [FIMA] [59]. Secondary parameters of PA include accelerometer-derived total energy expenditure as well as duration, percentage, and intensity of sedentariness, activity, and walking. Psychosocial evaluation of intervention effects include subjective age, HAPA-related questionnaires on intentions, action and coping planning, individual action control, sources of self-efficacy, outcome expectations, risk perception, and self-efficacy related to motivation, maintenance, and recovery [60]. Further, social support (Loneliness Scale [61]), habit strength (Self-Report Behavioural Automaticity Index; SRBAI [40]), and motivational quality (Behavioural Regulation in Exercise Questionnaire; BRE-Q-3 [62]) will be assessed. Exercise adherence will be part of the study procedure and an outcome measure in this trial. It will be assessed using the Exercise Adherence Rating Scale (EARS) [63] as part of the monthly-returned fall calendar. An additional evaluation of intervention sessions and assessment of psychosocial variables related to execution and habitualization of LiFE activities will be conducted during the intervention period.

#### Process evaluation

The MRC process evaluation framework emphasises the relations between *implementation* (what is implemented, and how?), *mechanisms of impact* (how does the intervention produce change?), *and context* (how does the context affect implementation and outcomes?) [31]. The process evaluation will complement evidence emerging from the trial. Quantitative measures used to assess these factors are marked in Table 4. Qualitative data will be gathered from focus groups which will be held within the study period. *Implementation* requires capturing whether the intervention was delivered as intended and in which quantity it was delivered. *Mechanisms of impact* will be explored using qualitative and quantitative outcome data related to the complex pathways leading to intervention-induced change. *Context* refers to external factors which may act as barriers or facilitators to intervention implementation, e.g., socioeconomic and social factors.

### Participant timeline

Screening procedure includes a telephone screening and a subsequent inhouse screening to determine eligibility. Telephone screenings will be performed to pre-screen potential participants regarding those in- and exclusion criteria that are measurable via telephone interview (see Table 4 for all variables). Those still eligible for participation will then be invited to the inhouse screening. In the inhouse screening, remaining exclusion criteria (labelled in Table 4) will be assessed to determine definitive eligibility for participation in the study. In case of medical uncertainties, the study’s medical advisor will be consulted; if the medical advisor is uncertain, the potential participant’s general practitioner will have to provide consent. Persons with a positive overall screening will then be scheduled for baseline assessment. As part of this assessment, participants will be equipped with a fall calendar for 1 year, an activPAL to assess PA for 9 days, and an activity diary to complement sensor-assessed PA measurement. After baseline assessment, participants will be randomized into either gLiFE or LiFE. The intervention is delivered as presented above. Post-assessments will be performed 6 months (follow-up 1; ± 2 weeks) and 12 months (follow-up 2; ± 2 weeks) after intervention start. All three assessments will last about 2 h each. Figure 1 gives an overview of the flow of participants.

Reasons for study drop-out or intervention drop-out will be recorded. In case of withdrawal from intervention, patients will still be eligible to participate in follow-up assessments given their consent. Reasons and date of withdrawal will be recorded in the database management system. Data recorded prior to withdrawal will be used unless the participant makes use of her/his right to have all data deleted.

### Sample size

The sample size was calculated using Pearsons’s Chi-square test, yielding *N* = 81 participants per group to be able to show noninferiority between treatments, accepting a non-inferiority margin of 20% difference to the reduction demonstrated in the original LiFE study [29]. Considering a drop-out rate comparable to the original LiFE study of 25% for all assessments [29], actually *N* = 108 participants per group are needed. To account for an additional variance due to the multi-centre design we increased the sample size by 10% and added another safety margin, coming out with an envisaged sample of a total of *N* = 300 community-dwelling participants to take part in the study: *n* = 150 at both study sites, i.e., the Network Aging Research (Heidelberg, Germany) and the Robert Bosch Hospital (Stuttgart, Germany).

### Recruitment

Participant recruitment started in April 2018; last participants will be recruited in June 2019. Participants will be recruited using data from the municipal registration offices in Heidelberg and Stuttgart. Recruitment will be performed via mail, with waves of 300 or 1000 letters being sent to randomly selected persons in the registry. In Heidelberg, residents from all city districts will be eligible to receive study information and a flyer with a prepaid response postcard to send back to the study centre if interested in participating in the study; in Stuttgart, due to the city’s much larger size compared to Heidelberg, residents from only one city district are contacted. To facilitate contacting the study centres, persons interested in the study may also use a contact form on the project website (http://www.life-alltagsuebungen.de) which was developed to enhance the project’s reach and public visibility. In addition, additional actions will be undertaken to support participant recruitment: In Heidelberg and Stuttgart, flyers and brochures will be distributed in pharmacies, physiotherapy and medical practices; press releases and articles will be launched in regional and district newspapers; lectures will be given at local public organisations concerned with health and aging. In Stuttgart, a cooperating health insurance company is going to contact their members in the desired age group living in the relevant city district via mail.

### Allocation and blinding

After completion of baseline assessments, participants will be randomised in either gLiFE or LiFE by the study coordinators in computer-generated blocks of variable size. To facilitate the gLiFE organisation process, all participants will be asked during the inhouse screening at which weekdays they have time to participate in a morning session (9.30 AM to 11.30 AM) or afternoon session (2.00 PM to 4.00 PM). gLiFE groups will be started once at least 8 and up to 12 persons are randomly allocated to this arm of the intervention and have a match in their schedule. LiFE participants can start the intervention immediately after the 9-day PA recording period has ended. All research staff will be eligible to perform telephone screenings, inhouse screenings, and baseline assessments prior to randomisation. Follow-up assessments after randomization will be performed by assessors blinded towards group allocation. To ensure blinding of assessors, the database used in this study will only show information not related to the intervention when assessors are logged in. For trainers and study coordinating staff, all information will be unlocked. Outcome measures which identify group allocation such as evaluation of intervention sessions will be collected by unblinded research staff.

### Data collection and management

A database management system will be used for data collection and management. Telephone and inhouse screening data will be directly entered into the database. Drawing items of the MoCA and the CES-D 10 will be performed on a paper sheet and item responses will be immediately entered into the database. For baseline and follow-up assessments, an electronic case report form (eCRF) will be used. Following the intervention sessions, machine-readable paper questionnaires will be used and personally transferred to the database manager on a regular basis. The database management system will set reminders for the collection of fall calendars, activity diaries, and activPALs: Once any of these items are overdue, the study coordinator will be notified. Participants’ individual identifiers and identifiable information will be kept on encrypted local servers at the two study sites as well as in the database, accessible only by authorized study personnel and—upon request—the external study monitor. Only research staff directly involved in data analysis will have access to the final dataset.

### Study monitoring

Quality assurance and control of the study will be performed by an external study monitoring institute being entirely independent of the coordinating investigator and institutions involved in the study conduct. This will include regular monitoring visits in every study centre, partial source data verification, continuous monitoring of the eCRF entries for 50% of the patients during the clinical part of the study, and checks for completeness and plausibility of eCRF.

### Statistical analyses

Statistical analyses will follow the ICH Harmonized Tripartite Guideline “Statistical Principles for Clinical Trials” E9 [64]. Based on the trial of Clemson et al., we expect a decrease in fall incidence (expressed as falls per amount of PA) of 79.4% for LiFE [29]. A one-sided Pearson’s chi-square test will be used to test noninferiority with α = 0.05 in the fall incidence between LiFE and gLiFE with a noninferiority margin of − 15.9%.

All main analyses will be done according to the intention-to-treat principle, which will include all randomized participants in the analysis dataset for whom a baseline assessment was conducted, regardless of their adherence to and compliance with the assigned treatment. Participants who withdrew or dropped out are requested to participate in follow-up assessments; those who are lost to follow-up will be included in the full analysis set by imputing their missing data. Secondary analyses will provide a more detailed insight on which dimensions both treatments are effective to deepen the understanding of trial results. Understanding the underlying physiological pathways in both formats will increase knowledge on the effectiveness of the intervention components and strategies and thereby improve the design and implementation of both LiFE treatments. Additionally, sensitivity analyses will be performed to explore the effect of missing values and attrition during treatment:

- Per-protocol analysis of the dataset restricted to all participants with available measurements for the primary variables and no protocol violations.
- If the number of missing values is substantial (higher than 5% for a given variable), a sensitivity analyses will be conducted for imputing missing values.

#### Data analysis and process evaluation

A detailed modelling of variations between participants and groups in terms of factors such as dose, acceptability, and contextual factors will be performed. Qualitative data will be interrogated using thematic analysis [65] in relation to its potential in organizing data following clear and concise guidelines with explicit stages, which will provide rich interpretation. We will integrate quantitative/qualitative process data into outcome datasets to examine whether effects on primary and secondary outcomes differ by implementation or contextual moderators. Quantitative and qualitative analyses will build upon one another (e.g., qualitative data will be used to explain quantitative findings and quantitative data will be used to test hypotheses generated by qualitative data), as specified by MRC guidelines [66].

### Dissemination

The main approach for scientific stakeholders will consist of scientific publications and conference presentations. Dissemination to end-users and the general public will be achieved through a project website (www.life-alltagsuebungen.de), containing information about the project, its progress, and results that are available to the general public. It contains links to other websites and social media to attract attention and to create awareness. After the end of the trial and in case gLiFE proves to be more cost-effective compared with LiFE, it is the aim to transfer and implement gLiFE into the healthcare sector in Germany. Steps included in this process will be a standardized curriculum as well as a LiFE trainer course.

## Discussion

Due to the high relevance and socioeconomic impact of falls in the older population, it is not surprising that numerous attempts based on various approaches have been made to reduce falls and fall-related consequences. The overall goal behind this is to optimize individual health trajectories in the aging process, which can be achieved by ensuring that community-dwelling older people receive high-quality, evidence-based fall prevention services [67]. Several systematic reviews and meta-analyses have been conducted in the field to identify best possible intervention measures, with somewhat diverse target groups and focusing on different intervention approaches. Exercise has emerged as a major cornerstone from these works. The most recent Cochrane systematic review on the topic had already found 159 trials with 79,193 participants back in 2012 [10], and concluded that multiple component group exercise and home-based exercise as well as home safety interventions reduce rate of falls and risk of falling. This is in line with other systematic reviews and meta-analyses, which found multifactorial interventions including exercise to be most effective, with exercise as a single intervention showing significant effects as well [67–69]. However, another recent Cochrane review showed that the majority of these multifactorial and multiple component studies are of low quality with unclear or high risk of bias [70]. Moreover, formal programs have largely failed to induce long-term behaviour change towards more regular exercise, often showing poor adherence (> 6 months) [16], and recent data do not demonstrate a reduction in the incidence of hip fractures in older adults [71]. Work on the effectiveness of exergaming has produced inconclusive results [72].

The LiFE program—as an exercise approach focused on the modification of individuals’ behaviour—has shown its effectiveness in a large randomised controlled trial, and it has been explicitly recommended for implementation as part of therapy practice to reduce fall risk in one of the aforementioned systematic reviews [67]. LiFE has shown its superiority relative to placebo and structured exercise [29], so assuming that LiFE *is* effective, the next step would be to look at its suitability for the recommended implementation on a large scale. Because LiFE requires seven individual one-to-one home visits, the program’s feasibility and affordability in everyday practice can be challenging. An economic evaluation of the LiFE program or a less costly alternative has never been conducted, despite the fact that the impact on both health outcomes and costs needs evaluating across competing interventions to enable well justified allocation decisions [52]. Therefore, a group-based LiFE approach should be tested for its noninferiority compared to the original, individually delivered LiFE program. Noninferiority trials aim to determine whether one treatment is not worse than a reference treatment by a predefined acceptable amount [32]. This kind of research is conducted on the premise that the “new” treatment has some other advantage compared to the reference treatment, for example less invasiveness and greater ease of administration [32]. In our study, gLiFE’s noninferiority in terms of fall incidence is evaluated with reference to the original LiFE program (LiFE): gLiFE will be recommended if it is not worse than LiFE by more than the predefined margin (∆ = 15.9%). As only a few studies of fall-related interventions have assessed costs and effects (e.g. in terms of QALYs) [70], and results were heterogeneous [73, 74], economic evaluations are needed to guide health care resource allocation.

Although Clemson and colleagues adhered to the common practice of evaluating fall prevention trials based on fall rates per total observation time (e.g., falls per person year) [29], in this study, we will estimate falls per risk exposure time, i.e., per walking duration and energy expenditure. Considering that, as the LiFE program promotes PA then it also enhances risk exposure of participants, the relationship of PA and falls might be a more adequate outcome rather than the rate of falls per total observation time [26, 75]. This is more likely an appropriate analysis considering that both physical inactivity and high falls risk are negatively associated with healthy life expectancy [76].

As a limitation, it has to be acknowledged that in such a trial, it cannot immediately be distinguished between effective and ineffective treatment due to the lack of (placebo or “sham”) control, i.e., both treatments/interventions could be ineffective [77]. However, the LiFE study by Clemson et al. has shown its high value and effectiveness, even though in only one large trial.

Concluding, this study will be of high practical relevance as noninferiority of gLiFE would facilitate nationwide implementation due to lower financial and personnel requirements. This is of special importance in the face of the large number of older people in need of preventive measures which have to be provided by a small number of therapists, especially when it can be expected that the ratio of therapists to patients will increase. Due to the lack of knowledge on the participants’ personal (home) environment and the consequential limitation of individualization options of LiFE activities in the group (gLiFE), we do not expect the group format to be superior to LiFE. However, LiFE activities are graded and individually adapted to each gLiFE participant’s functional level, also taking into account personal routines and environment as described by the participant, and using simulated demonstration instead of demonstration in the actual situation as provided in LiFE. Unlike LiFE, utilizing LiFE in a group may also enrich the program’s prosperity through psychosocial resources such as social interaction and mutual role-modelling of the participants [78, 79].

### Trial status

The study is still ongoing; we expect to enrol the last participant by July 2019. Study completion date will be September 30th 2020. By the time of submission (September 26th, 2018), *n* = 127 participants were already enrolled in the trial.

## Abbreviations

ABC Scale: Activities-specific balance confidence scale
BRE-Q-3: Behavioural Regulation in Exercise Questionnaire
CES-D 10: Center for Epidemiologic Studies Depression Scale, 10 item Version
EARS: Exercise Adherence Rating Scale
eCRF: Electronic case report form
FES-I: Falls Efficacy Scale International
FIMA: questionnaire for the use of medical and non-medical services in old age
gLiFE: Group-based lifestyle-integrated functional exercise
HAPA: Health Action Process Approach
ICERs: Incremental cost-effectiveness ratios
KIT-FG: Cohesion in teams–leisure and health sport
LiFE: Individual lifestyle-integrated functional exercise
LiFE: Lifestyle-integrated functional exercise
LLFDI: Late-life function and disability instrument
MoCA: Montreal cognitive assessment
MRC: Medical Research Council
PA: Physical activity
QALYs: Quality-adjusted life years
SRBAI: Self-report behavioural automaticity index
TIDieR: Template for intervention description and replication
